# A Narrative Review of Kangaroo Mother Care (KMC) and Its Effects on and Benefits for Low Birth Weight (LBW) Babies

**DOI:** 10.7759/cureus.31948

**Published:** 2022-11-27

**Authors:** Mithila Koreti, Pramita Muntode Gharde

**Affiliations:** 1 Community Medicine, Jawaharlal Nehru Medical College, Datta Meghe Institute of Medical Sciences, Wardha, IND

**Keywords:** kangaroo mother care, accredited social health activist workers, father's role, nurses, family, breastfeeding, infant, skin-to-skin contact, low-weight babies

## Abstract

Kangaroo mother care (KMC) is a preventative, economical method for infants with low birth weight (LBW). KMC benefits LBW infants in a number of ways. This review standpoints the effect of KMC on the weight gain of LBW neonates. KMC also improves breastfeeding rates during the hospital stay as well as at home. KMC can be provided not only by mothers but also by fathers and other adults in the family. However, it is not routinely practiced in hospitals. Short-term and long-term KMC is beneficial for survival, neurodevelopment, breastfeeding, and mother-infant bonding. Preterm infants are more likely to experience neonatal mortality and morbidity due to acute breathing problems, gastrointestinal problems, autoimmune disorders, and neurological defects as compared to full-term and normal-weight infants. A thorough literature search was conducted using key databases like PubMed and Google Scholar, as well as Medical Subject Heading (MeSH) terms and related keywords. Clinical health experts also believed that implementing KMC would assist mothers in developing more solid emotional bonds with their newborns. As a result, both mothers and their newborns felt more secure, and the babies were more relaxed. KMC was also seen to support the infants' growth and development, which improved the mothers' sense of connection. It is crucial to remember that KMC works better for babies with very low birth weight (VLBW). The mother-child relationship enhances sucking-feeding, and KMC infants have higher means of growth parameters.

## Introduction and background

The WHO defines low birth weight (LBW) as less than 2,500 gm at birth, very low birth weight (VLBW) babies are those who weigh less than 1,500 gm, and extremely low birth weight (ELBW) babies weigh 1,000 gm [1]. A new approach called kangaroo mother care (KMC) was initiated to provide care to LBW babies. It promotes optimal heat management, breastfeeding, infection prevention, and bonding, all of which benefit their health and well-being. In KMC, premature infants are kept skin-to-skin contact with their mothers. It is a straightforward and efficient method for enhancing the health and development of both early and full-term infants. KMC was first instigated by Dr Edgar Rey and his Colombian pediatrician colleagues in the late 1970s [2].

It primarily devised strategic means to compensate scarcity of facilities for treating LBW babies [3]. KMC shortens hospital stays and lowers rates of hypothermia and neonatal infections. KMC can be provided in settings with limited resources because it does not require technology or electricity [4]. LBW results from premature birth and constrained intrauterine growth. It is linked to escalating fetal or neonatal mortality rates, slowed growth or cognitive development, and later-life chronic diseases. One of the major goals of "A World Fit for Children" is to reduce LBW incidence by about one-third between 2000 and 2010. In 2002, the United Nations General Assembly Special Session on Children adopted the action plan and declaration for this goal, hence, encouraging bonding and breastfeeding [5]. KMC is adopted worldwide by placing the premature newborn against the mother's chest to promote bonding and breastfeeding [6].

Clinical health experts also believed that implementing KMC would increase mothers' emotional bonds with their infants. As a result, both mothers and their infants were calmer and newborns felt safer. KMC was also observed to promote the infants' development and growth, which resulted in the mother feeling a stronger sense of connection [7]. KMC does not require equipment use. The KMC technique helps in providing care to premature infants and delivers increased protection to the baby [8].

KMC involves skin-to-skin contact between the baby and the mother. Skin-to-skin contact between the mother and baby begins at birth and persists constantly till the baby is stabilized. KMC continues for at least one-to-three hours every day and generally takes three-to-seven days. For instance, when the baby is being bathed, there might be brief breaks. One hour after giving birth, the mother has to start breastfeeding and it might last for two to three hours. The mother must be trained to express milk so she can feed the baby from a cup as an underweight newborn is too weak to suck properly in the initial few days. When the mother is busy with her daily tasks, other family members can give the newborn KMC as well. The baby experiences weight gain and there are increased number of hours for sleep due to KMC [5]. The skin-to-skin contact method involves placing the baby on the mother's bare chest, usually covered with a warm blanket [9].

In the long run, more than two out of every three premature babies will have no developmental issues. In contrast, most others will have only minor to moderate disabilities or delays. This photosynthetic therapy will help preventing complication due to physiological jaundice. More intensive therapies like blood exchange transfusions or intravenous antibody injections may be necessary if phototherapy therapy is not successful, but these are rare. Common health problems in children include LBW, malnutrition, childhood infection, disability, and deformity. The scope of the issue and the best care for LBW/preterm infants should be discussed and incorporated into ongoing community activities. Fathers or family members who have successfully cared for LBW/preterm infants using KMC [10]. In order to fulfil their developmental support, full-term infants and premature infants are given KMC for six months and three months, respectively. The infant is wrapped in cloth, and the adult and newborn are in skin-to-skin contact on the chest. Baby wearing is a common practice that can be done with various carriers and slings. The child may be in front of or behind the adult, and they are both fully dressed [11].

Only a small percentage of LBW infants can receive medical attention in hospitals in most developing nations. In these environments, some births still occur at home, and newborns delivered in facilities are often released before their due dates. Therefore, it is essential to assess KMC in a thorough and efficient manner [12]. Although these complications are not identified until late in the hospitalization, it has been discovered that providing parents with a comprehensive view of the hospitalization is beneficial. LBW babies have difficulties within the first 24 hours of birth. All newborns who weigh less than 2,500 gm are qualified for KMC. There are more than 20 million babies worldwide, or 15.5% of all births, who have LBWs. According to WHO, the annually incidence of 33% of babies born in India are underweight. The leading and second-leading causes of newborn deaths worldwide are both prematurity [12].

According to Indian scientists, KMC is the term used to describe the care given by a newly delivered mother to her LBW newborn baby by placing the baby between her breasts. The baby is kept alive by the mother in direct skin-to-skin contact [13]. The benefit to the mother comes from her keeping the baby warm. It boosts the mother's confidence in her ability to care for her newborn and increases the amount of breast milk she produces. Assistance with positioning, feeding, infection control, and breathing problems is given both during hospitalization and after an early discharge. Skin-to-skin contact should be introduced gradually in the nursery to ensure a seamless transition from initial care to ongoing KMC. Sessions that last less than an hour should be avoided because the baby might get stressed out from the constant handling. Skin-to-skin contact should instead increase and continue throughout the day. There must be at least six to eight hours of practice [13].

KMC provides infants tactile stimulation by facilitating early skin-to-skin contact between mother and infant. In addition, it stimulates their kinetic, olfactory, and afferent sensory systems through breastfeeding and nipple sucking. In addition, it improves bonding, attachment, and interaction between mother and child, which is essential for the social and emotional development of the child. This review article aims to investigate the effects of KMC on newborns with LBW. To determine the therapeutic effect of maternal care on LBW infants [14].

## Review

An online search was conducted using PubMed, Google Scholar, and Web of Science to find scholarly articles. Key Medical Subject Heading (MeSH) search terms such as "Kangaroo mother care benefits," "Low birth weight baby," "neonatal child," "preterm child," and "infant child development" were used. They were used interchangeably and in combination to find all relevant articles. The literature search used the boolean terms AND, OR, and BUT for more clear results. 45 articles were retrieved and 36 out of those were utilized for this review based on the articles’ relevance. All articles with free and full text availability through PubMed were included in this review. The articles ranging from 2000 to 2022 were used, highlighting the essence of breastfeeding and why it is important. The emphasis of this review is on the importance of KMC for newborns with LBW. Case reports were not evaluated in the review.

KMC and LBW babies

KMC and LBW babies’ discussions about the severity of the issue and appropriate care for LBW/preterm infants are being incorporated into already existing community activities. An efficient strategy for enhancing the well-being of LBW infants is KMC. It is a simple approach that does not require trained personnel and can be practiced even in remote health centers and continued at home. It is beneficial not only to the baby but also to the mother [13]. KMC services have primarily been made available in tertiary facilities [15]. Earlier, the most common problems of hypothermia and infection in newborns was because of the lack of practice and implementation of KMC [16].

KMC can explain most of the mothers' positive attitudes in strengthening the interaction and bond between the mother and her newborn. However, the "uncomfortable factors" that were noted included the concern about heat, sweating, and discomfort faced by more than half of the mothers [17]. At the national level, KMC should be integrated into core neonatal, maternal, and child health policies, with appropriate monitoring, assessment, and evaluation [18].

Extra research is necessary to confirm the effects of KMC on infant development and physiologic stability. Infants from the KMC had larger heads, were heavier, and performed better in neurobehavioral development. Infants who are clinically stable, preterm, or extremely preterm can maintain a stable body temperature, heart rate (HR), respiratory rate (RR), and oxygen saturation rate (O_2_ sat). Infants maintain stable body temperature during KMC in the early postpartum period [19]. KMC lengthens sleep, including periods of quiet rest [20]. Most clinical providers learned about KMC through personal observation or experience with KMC's practice. Many people had no formal training they lacked confidence in treating LBW with KMC, which also had to do with newborn safety [21].

KMC is advantageous because it strengthens the mother-child bond, assisting LBW babies in stabilizing HR. This was found in certain studies where LBWs were observed and mothers were asked to assist in implementing KMC while hospitalized. Furthermore, clinical providers believed that the mother and their new born placement in KMC are beneficial for faster weight gain due to the LBW baby's easy access to breast milk [22]. KMC encourages bonding, stimulates breast milk production in the mother, and brings the milk source closer to the infant and be more accessible [23].

Furthermore, KMC is favorable in improving baby's breathing pattern, making the breathing more regular and decreasing crying. Clinical health experts also believed that implementing KMC would increase mothers' emotional bonds with their newborn's as a result, both mother and their newborns felt more secure, and the babies were calmer. The mother felt a stronger connection to her children because of how KMC was seen to support their growth and development [7]. KMC is a feasible and acceptable procedure, and mothers of LBW babies had high adoption rates [24]. 

KMC in Neonatal ICU (NICU) Care

If possible, deliveries should be done in an ICU of a good medical hospital with a high-risk maternity unit and an NICU. LBW babies in hospitals may get care like being put in temperature-controlled beds and getting fed through an IV or a Ryle's tube if they cannot breastfeed. LBW infants who are qualified for "KMC uniform hypothermia" should be treated in critically ill newborns who require special attention and have a body temperature below 36.5°C. Another issue to consider in the care of premature babies is oxygen saturation. KMC entails infant and direct and ongoing skin-to-skin contact between mother and child. It aids in the prevention of growth retardation, the improvement of weight gain, and the reduction of infection [25]. In neonatal care facilities, unwell and young babies receive treatment in the NICU, which is staffed 24 hours a day, seven days a week [26]. 

KMC and Accredited Social Health Activists (ASHAs)

We suggest that rather than community-level health workers like ASHAs, the beginning of KMC requires a worker's involvement. ASHAs might offer aftercare support. Local employees similar to auxiliary nurse mid-wives (ANM) can also help in providing the required intervention. If professional trained staff are available, they can also help. The ANM is present when issues arise, and skilled community health workers, who function as the equivalent of government ASHAs, offer follow-up care. All these health workers are introduced to KMC, which is relatively new and can be used in a community setting [12].

A KMC session should last at least one hour because frequent handling can be stressful for the neonate. The newborn recognizes the smell of the mother’s milk, which activates the reflexes of the neonate and helps in the development and growth. The presence of ASHAs can note the apprehension as well as how the mother is handling her child. Each KMC session should be gradually increased for as long as the mother can comfortably provide. Studies have shown that after just four weeks, KMC reduces stress, anxiety, and depressive symptoms in preterm baby mothers. ASHAs can further counsel them whenever required [27]. For preterm or LBW newborns, KMC is a well-recognized option for treatment based on evidence that KMC relies heavily on the mother and child making skin-to-skin contact. The WHO advises that KMC be given as standard therapy to newborns weighing 2,500 gm or less at birth and that it be started in medical facilities as soon as the infants are clinically stable to improve preterm birth outcomes [28].

Aside from disparities in attitudes toward KMC, several impediments have been identified. The safety of facilitating KMC in the preterm infant is a frequently mentioned barrier. In particular, the intubated newborn pose a limiting opportunity to provide KMC and this is depicted in several studies [29]. KMC is placing a snuggled baby on a parent's chest while holding the child upright and in ventral skin-to-skin contact to simulate marsupial care [23]. This technique must be introduced to every new parent after birth by any health worker or ASHA.

KMC as a Gold Standard of Care for All Infants

It is strongly suggested that KMC become the gold standard of care for providing high-quality healthcare to all infants, regardless of location or socioeconomic status, as a practical, natural, and affordable intervention. The implications of its use as a successful care for the neonates. Irrespective of a newborn's location or financial situation, it is suggested that KMC be used as a standard of care because it is a practical, organic, and economical intervention. Its use has been testified to improve neurodevelopment, decreased mortality, physiological domains like temperature regulation, and cardiovascular stability, along with certain behavioral domains like sleep and nursing duration and, hence, is proved to be an effective therapy for neurodevelopment. In addition, it is also an effective way for overall development of a new born.

KMC use in clinical practice is inconsistent and underutilized. Furthermore, whether continuous KMC should be advised in all circumstances or if there is a crucial start, dose, or ideal duration window is not clear. This review summarizes the most recent research on the advantages of KMC for preterm infants, focusing on the distinctions and similarities between low- and high-resource nations. Additionally, there are implementation considerations and unanswered issues that require further inquiry. Mothers expressed concerns for their children's safety during KMC, including worries that the child might pass away. It is important that the therapy is more available to preterm children, even though they suggest many obstacles to a successful KMC implementation. Educational interventions state that personnel must receive training and the knowledge and abilities required to support and aid KMC properly [30]. Depression, mental diseases, drug addiction, and infections are some of the maternal concerns [29].

Regardless of birth weight, gender, or delivery method, intermittent KMC with more nursing opportunities was found to help promote neonatal weight gain. In light of this literature review, KMC should be regarded as an efficient method for accelerating weight gain in newborns [31]. By providing fathers with KMC opportunities, they could take on their fatherly responsibilities and deal with unforeseen circumstances. The physical setting and contradictory staff reports affected their capacity to care for their preterm newborn and their experiences [32].

The benefits and efficiency of KMC as an alternative procedural pain control technique remained consistent. There is a need for research into overcoming the barriers that prevent people from using KMC during and after painful procedures [33]. KMC can be promoted during prenatal care, visits to expectant mothers' homes, and in women's groups. This can be done by community health and extension workers, midwives, and women with experience using KMC [15]. According to our review, skin-to-skin contact is the minimum essential element of KMC, and variations depending on the situation and the unique clinical requirements of the newborn [34].

**Figure 1:**
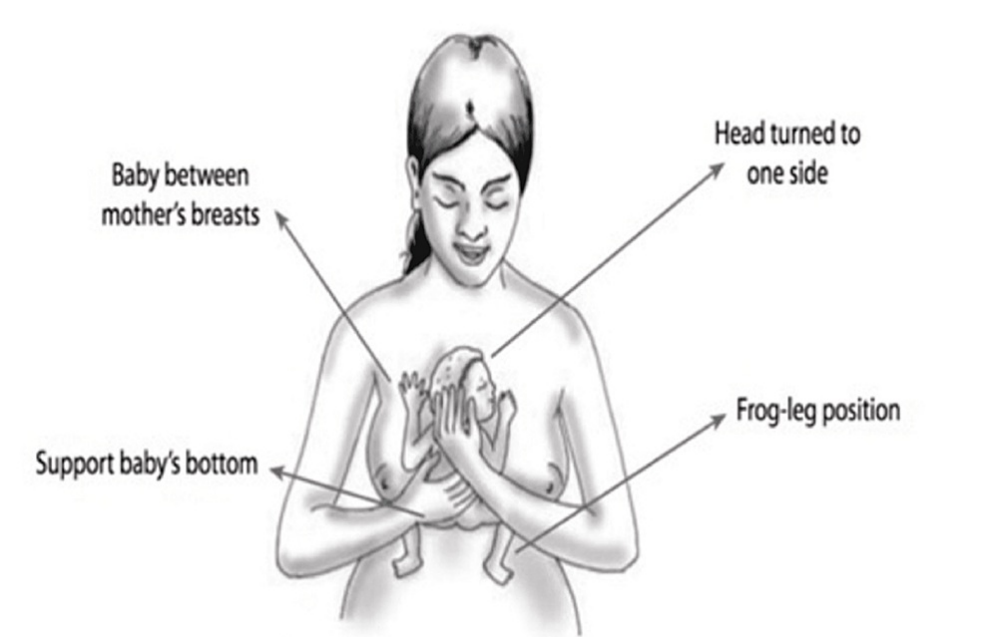
Kangaroo mother position: how to hold the infant in KMC Source: Jadhao A et al. [2] KMC: Kangaroo mother care

KMC can be promoted during pregnancy as health education to the going-to-be-mothers, midwives, community health workers, and women with prior KMC experience can all perform this and set example for the new mothers [35]. Additionally, delaying the start of continuous KMC until the infant is stable is advised. This involves waiting until the child can breathe on its own without the assistance of additional oxygen. KMC should be initiated in hospitals for routine care of preterm birth babies weighing 2,500 gm or less until they are clinically stable. Families' attitudes toward their involvement with KMC and overall positivity were reported by health professionals as being favorable [36]. Figure 1 depicts how to hold the infant in KMC.

Limitations

A disadvantage of intermittent KMC is that it involves less frequent breastfeeding than needed. KMC must be maintained daily. It is acceptable to lessen skin-to-skin contact and increase the frequency of breastfeeding if the infants refuse to be put in the kangaroo position as they become older. Fathers or other family members can alternately provide skin-to-skin contact to give mothers a break.

## Conclusions

KMC provides stable LBW infants with the best conditions for growth, boosts parental involvement and empowerment, and aids in the healing process. It is a scientifically sound, effective, and efficient substitute for neonatal care units in many settings, despite relying on straightforward interventions. It provides high-quality care at a fraction of the cost of traditional care, and it raises both mother's and baby's satisfaction. KMC should be implemented as soon as possible because it helps families and the community get ready for a successful hospital discharge while letting parents continue to be the primary direct caregivers for LBW infants in both wealthy and underprivileged environments. The review suggests that KMC may also lower morbidity and hospital stays in underdeveloped areas. Also, it is an effective and low-cost technique that protects newborns from hypothermia and promotes child growth. KMC boosts the mother's confidence in caring for her preterm infant.
